# Linitis Plastica-like Metastases to the Gastrointestinal Tract on Cross-Sectional Imaging

**DOI:** 10.3390/biomedicines13092197

**Published:** 2025-09-08

**Authors:** Ana Veron Sanchez, Elena Canales Lachen, Maria Gomez Galdon, Luigi Moretti, Calliope Maris, Ana Maria Bucalau, Charif Khaled, Maria Antonietta Bali

**Affiliations:** 1 Department of Radiology, Institut Jules Bordet, Hôpital Universitaire de Bruxelles, 90 Rue Meylemeersch, 1070 Brussels, Belgium; 2 Department of Radiology, Hospital Universitario Ramon y Cajal, Ctra. de Colmenar Viejo km. 9100, 28034 Madrid, Spain; 3 Department of Pathology, Institut Jules Bordet, Hôpital Universitaire de Bruxelles, 90 Rue Meylemeersch, 1070 Brussels, Belgium; 4 Department of Radiotherapy, Institut Jules Bordet, Hôpital Universitaire de Bruxelles, 90 Rue Meylemeersch, 1070 Brussels, Belgium; 5 Department of Gastroenterology, Institut Jules Bordet, Hôpital Universitaire de Bruxelles, 90 Rue Meylemeersch, 1070 Brussels, Belgium; 6 Department of Digestive Surgery, Institut Jules Bordet, Hôpital Universitaire de Bruxelles, 90 Rue Meylemeersch, 1070 Brussels, Belgium; charif.khaled@hubruxelles.be; 7 Department of Radiology, Agostino Gemelli University Polyclinic, Largo Agostino Gemelli, 8, 00136 Rome, Italy

**Keywords:** linitis plastica, gastrointestinal metastases, malignant target sign

## Abstract

This review provides an overview of the cross-sectional imaging features of gastrointestinal (GI) metastases presenting with a linitis plastica (LP) pattern and illustrates these findings through a series of cases from various primary tumors. It also addresses key diagnostic challenges, with particular attention to differential diagnosis. The term linitis plastica (LP) refers to the macroscopic appearance of a hollow organ with diffuse mural tumor infiltration, leading to loss of parietal distensibility. Although rare, primary LP can occur throughout the gastrointestinal (GI) tract. First described in the stomach—the most common site—it is typically associated with undifferentiated adenocarcinoma composed of poorly cohesive cells, often with signet ring morphology. Beyond primary GI tumors, LP-like metastases may also arise from extragastrointestinal primaries, most notably breast carcinoma (particularly the lobular subtype), as well as urinary bladder and prostate carcinomas. LP-like GI metastases typically manifest as circumferential, enhancing wall thickenings with exaggerated zonal anatomy and luminal narrowing. Due to diffuse parietal tumor infiltration—often with mucosal preservation—the submucosa and serosa appear disproportionately thickened and show greater enhancement relative to the muscularis propria (MP). This specific imaging appearance is known as the malignant target sign, which must be distinguished from the benign target sign, where the most prominent low-density layer corresponds to edematous submucosa. Additional key features include homogeneous enhancement with loss of layer differentiation on delayed-phase imaging and a concentric ring pattern on MR. Secondary findings may also be present, such as intestinal obstruction and concomitant peritoneal carcinomatosis (PC). Gastrointestinal metastases with an LP pattern present a significant diagnostic challenge, as they can mimic both primary tumors and benign inflammatory or infectious conditions. Accurate diagnosis is critical because management strategies differ substantially. Since the mucosa is often spared, endoscopy and superficial biopsies may yield false-negative results. Therefore, while immunohistochemistry (IHC) remains essential for confirmation, radiologists play a pivotal role in raising suspicion for LP-like GI metastases and recommending deep, extensive biopsies to obtain adequate representative tissue. Furthermore, in cases of an unknown primary tumor, recognition of the LP pattern can provide important clues to the potential site of origin.

## 1. Introduction

Metastases to the gastrointestinal (GI) tract have traditionally been regarded as rare. However, the relatively high incidence reported in autopsy studies—for example, up to 35% in patients with breast cancer—suggests they may be more common than previously thought [1]. Within this context, a distinct subgroup with LP-like features has also been described [2]. The term LP-like refers to its resemblance, on cross-sectional imaging, to primary LP. The concept of LP was first introduced in 1779 to describe a gastric condition marked by scirrhous tissue [3]. Its name derives from the characteristic bands of fibrous tissue within the submucosa, resembling linen fibers [4]. Over time, various definitions emerged [4,5] until, in 1953, LP was established as a gastric cancer subtype characterized by abundant fibrous stroma and associated with a poor prognosis [6,7].

The diagnosis of LP-like metastases to the GI tract poses significant challenges throughout the diagnostic process. On cross-sectional imaging, reaching the correct diagnosis is often difficult, as an isolated GI metastasis is less likely than a second primary tumour [8]. Moreover, LP-like metastases can closely mimic various benign conditions, further complicating interpretation. Nonetheless, establishing the correct diagnosis remains essential, as therapeutic strategies and prognosis may differ substantially.

To the best of our knowledge, no series has yet been published describing LP-like metastases to the GI tract arising from different primary tumours. The purpose of this work is to illustrate the cross-sectional imaging features of this distinct metastatic presentation, supported by a review of the literature. In addition, we discuss key points for differentiating this entity from other malignant and benign conditions that may present with similar imaging characteristics.

## 2. Primary Gastrointestinal LP

Since LP-like metastases closely mimic primary LP, we will first provide a concise overview of gastric LP, followed by a description of their key imaging characteristics.

### 2.1. Primary Gastric LP

Primary gastric tumors arise from fundic gland cells within the deep mucosa and spread centrifugally into the submucosa, muscularis propria, and serosa, accompanied by a desmoplastic reaction [9]. This process leads to diffuse infiltration of the gastric wall, where tumor cells are dispersed within the stroma rather than replacing it, unlike mass-forming tumors. As a result, the stomach retains its layered architecture but appears markedly thickened due to the combined effects of deep infiltrative growth and prominent desmoplastic response Although often used interchangeably with terms such as scirrhous, Lauren, or signet ring cell carcinoma, LP does not correspond to a single pathological entity and should instead be reserved for describing the macroscopic characteristics of the tumor [10,11,12,13]. It is most commonly associated with poorly differentiated adenocarcinoma composed of poorly cohesive cells that stimulate fibroblast activation [4,14]. This accounts for the pronounced desmoplastic reaction, resulting in a rigid, contracted stomach with marked luminal narrowing and the classic ‘leather bottle’ appearance [15]. Signet ring cells—characterized by abundant cytoplasmic mucin and an eccentrically displaced nucleus [14]—may also be variably present.

As early as 1933, the mucosa was described as being either preserved or only minimally affected [16]. More recently, advanced endoscopic techniques have revealed abundant, though abnormal and non-functional, neovascularization, thought to result from the mechanical compression caused by extensive reactive submucosal fibrosis [17]. In later stages, rugal folds may disappear due to rigidity and mucosal flattening, or alternatively become thickened and swollen, creating a characteristic waffle-like pattern [18].”

#### Primary LP GI Tumours: Cross-Sectional Imaging

**Figure 1:**
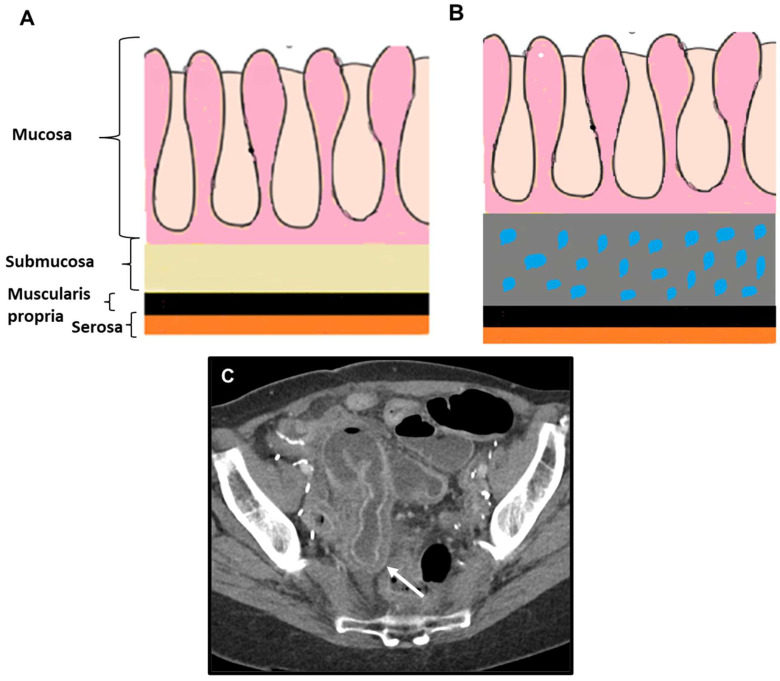
(**A**) Diagram of normal layers of the gastrointestinal tract. (**B**) Schematic illustration of the target sign in benign conditions, where the submucosa appears thickened and constitutes the most prominent low-attenuation layer due to edema (blue dots), inflammatory infiltration, blood products, or fat. (**C**) Axial contrast-enhanced CT showing a benign target sign (arrow) in a case of radiation-induced enteritis.

On cross-sectional imaging, gastric LP typically presents as a diffusely infiltrated stomach with symmetric concentric wall thickening and a characteristic accentuation of the normal layered anatomy. Additional hallmark findings include fold effacement, gastric wall rigidity, and reduced distensibility. The concentric thickening of the gastric wall resembles the ‘target sign’ described by Balthazar on contrast-enhanced CT (CE-CT) in inflammatory and ischemic bowel disease (Figure 1). This sign is characterized by three concentric rings of alternating attenuation: high–low–high. The hyperattenuating layers represent the mucosa and serosa, while the hypoattenuating ring corresponds to the submucosa, which appears most prominent due to edema, inflammatory infiltration, blood products, or fat [19,20]. The target sign has been shown to aid in distinguishing benign from malignant conditions [21]. Nevertheless, scirrhous gastric tumors have been noted as an exception, as they too may display this feature [21,22].

**Figure 2:**
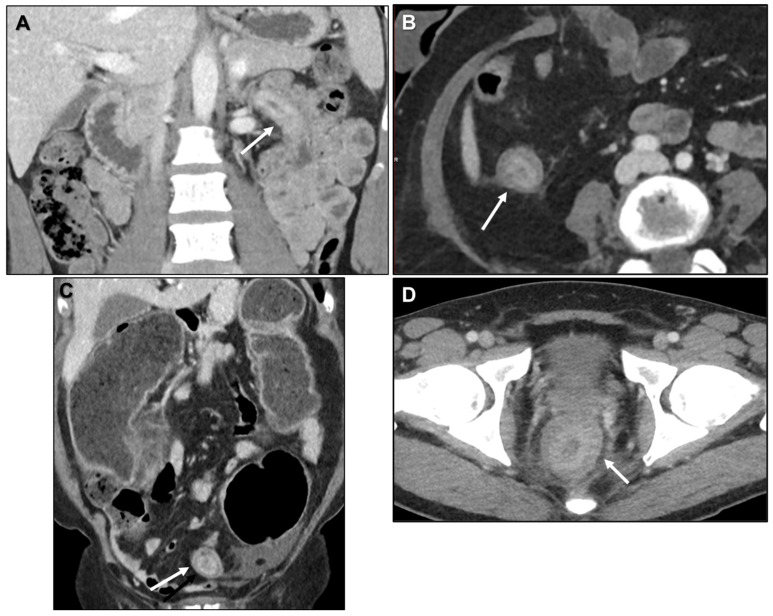
Primary gastrointestinal tumours with a linitis plastica pattern. These lesions demonstrate tumour infiltration involving the full thickness of the bowel wall, while maintaining the layered architecture, ultimately resulting in a rigid and contracted bowel segment. (**A**) Coronal contrast-enhanced CT (CE-CT) shows an undifferentiated tumour of duodenum (arrow). (**B**) Axial CE-CT depicts an undifferentiated tumour of ascending colon (arrow). (**C**) Coronal CE-CT reveals an undifferentiated tumour of sigmoid colon (arrow). (**D**) Axial CE-CT demonstrates an undifferentiated tumour of rectum (arrow).

Besides the stomach, undifferentiated and poorly differentiated primary tumors of the small bowel (SB) and colorectum may also present with a LP pattern [10,23,24,25,26,27,28,29]. On cross-sectional imaging, these typically appear as tumor infiltration of a relatively long bowel segment (>5 cm), involving the full thickness of the wall while preserving its layered structure, ultimately resulting in a rigid and contracted segment [24] (Figure 2).

### 2.2. LP-like Metastases to the GI Tract: Primary Tumours

LP-like metastases to the GI tract are more common than primary LP tumors [30,31]. In general, metastases from a primary GI LP display similar imaging characteristics [31,32,33]. However, several non-gastrointestinal primaries have also been reported to metastasize to the GI tract with this pattern.

Breast cancer is the second most common primary tumor to metastasize to the gastrointestinal tract, after melanoma [34]. Although invasive lobular carcinoma (ILC) represents only 5%–15% of all breast cancers [35], it is the subtype most frequently associated with GI metastases, with an incidence of up to 40% compared to just 2% for invasive carcinoma of no special type (NST, the current term for invasive ductal carcinoma) [36]. ILC metastases to the GI tract often present with an LP pattern, reflecting the diffuse infiltrative growth seen within the breast [37,38]. This characteristic behavior is linked to genomic alterations leading to loss of E-cadherin, the adhesion molecule responsible for cell-to-cell cohesion [39]. While the stomach is the most commonly affected hollow organ [38,40], the rectum, colon, and small bowel may also be involved. Reported incidence rates vary, likely reflecting the limited sample sizes of published series [37,40,41].

LP-like metastases to the GI tract from prostate or bladder cancer are much less common, with only 13 cases of rectal involvement reported for both tumors combined [42]. Even more rarely, lung, pancreaticobiliary, and ovarian carcinomas may metastasize with an LP pattern [20,43,44]. Although gallbladder carcinoma has traditionally been included in this group, no published case reports have been identified to date.

#### 2.2.1. LP-like Metastases to the GI Tract: Imaging Features

Regardless of their origin and their location, LP-like metastases have been reported to show the same imaging characteristics (Table 1). Unlike mass-like metastases, they appear more infiltrative as regular, symmetrical and concentric thickened segments with a narrowed lumen [31,32].

**Figure 3:**
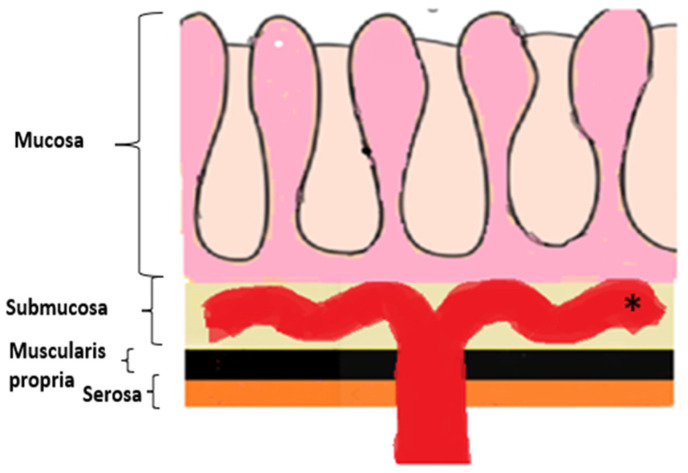
Schematic representation of the layers of the gastrointestinal tract and their associated vascular supply. The diagram illustrates the mucosa, submucosa, muscularis propria (MP), and serosa, together with the corresponding vascular networks. Most of the arterial branches, venous channels, and lymphatic vessels that provide perfusion and drainage to the mucosa and MP arise from interconnected plexi located within the submucosa (*).

Understanding the dissemination pattern of GI LP-like metastases is essential for accurate interpretation of imaging features. The submucosa is typically the most affected layer and serves as the primary site of involvement. This is likely due to the predominantly hematogenous route of spread, as well as the fact that most arterial, venous, and lymphatic vessels supplying the mucosa and muscularis propria arise from plexuses within the submucosa (Figure 3). Once metastatic deposits reach this layer, they may extend circumferentially and continuously via the lymphatic channels [1]. An alternative route is longitudinal intramural spread, facilitated by the peristaltic and antiperistaltic “milking” forces of the bowel wall [45].

In our clinical practice, CT images are acquired on the axial plane at the portal venous after intravenous administration of iodine contrast medium, with a flow rate of 3 mL/s. Multiplane reconstruction can be obtained on coronal and sagittal planes. No oral contrast is required. When using a dual-source scanner, virtual, unenhanced images can be generated. MR images can be obtained using 1.5 T or 3.0 T field-strength MR scanners with a surface-phased array coil covering the abdomen and the pelvis. The acquisition protocol includes coronal and axial T2-weighted images and diffusion-weighted imaging (respiratory-triggered for the upper abdomen and on breath hold for the inferior abdomen) with 3 b values of 0, 150, and 800 s/mm^2^. Following intravenous injection of 0.2 mmol/kg of gadolinium-based contrast agents, fat-suppressed 3D T1-weighted sequences are obtained upon breath hold at the arterial and portal phases covering the upper abdomen and after a 3 min late phase of the whole abdomen.

##### Malignant Target Sign

**Figure 4:**
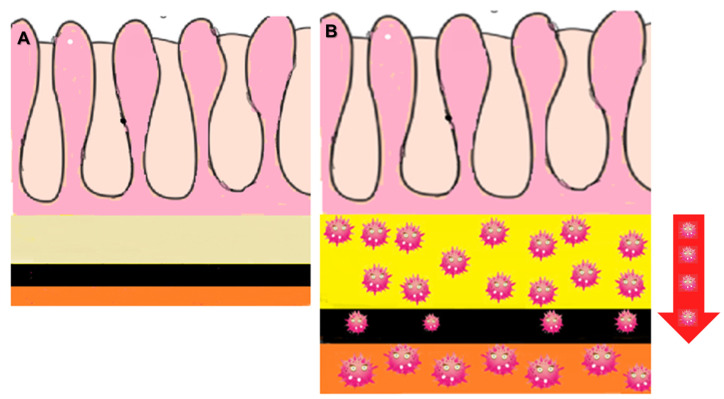
(**A**) Scheme of the layers of the normal GI tract. (**B**) Scheme representing the centrifugal infiltration of LP-like metastases within the bowel wall. The submucosa is usually the most affected layer, as it represents the initial site of metastatic infiltration and is composed of loose connective tissue. As the tumor spreads centrifugally, it next involves the muscularis propria (MP), where the dense arrangement of muscle fibers makes infiltration less pronounced than in the submucosa. The subsequent layer affected is the serosa, which consists of vascularized connective tissue that is more distensible than the MP.

**Figure 5:**
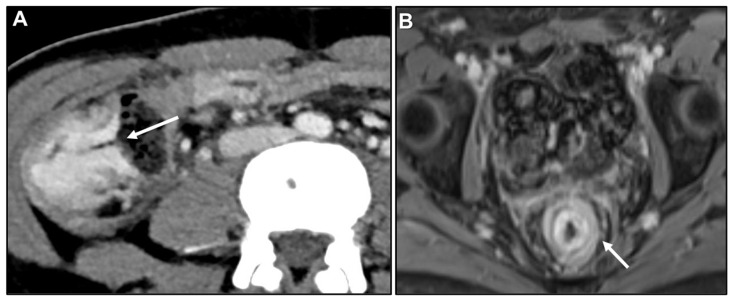
Malignant target sign. (**A**) Axial contrast-enhanced CT. Linitis plastica-like metastasis to the caecum from invasive lobular carcinoma (arrow). (**B**) MR image: Axial CE-GRE-T1WI. Linitis plastica-like rectal metastasis from breast adenocarcinoma (arrow).

This imaging feature is a distinct variant of the benign “target sign” and is highly suggestive of LP-like metastases on CE-CT [20,44]. The submucosa is typically the most affected layer, as it represents the primary site of metastatic infiltration and contains loose connective tissue. These features make it the layer with the highest tumor burden, resulting in marked enhancement. As the tumor spreads centrifugally, it reaches the MP. Because this layer consists of densely packed muscle fibers, infiltration is less pronounced compared to the submucosa. The serosa is usually involved next; it is composed of vascularized connective tissue that is more distensible than the MP (Figure 4). After intravenous contrast administration, this pattern produces the characteristic “malignant target sign” of metastatic involvement of the GI tract: a markedly thickened and enhancing submucosa, a relatively less thickened and less enhancing MP, and a variably thickened serosa with greater enhancement than the muscularis propria (Figure 5). However, it should be emphasized that the “malignant target sign” is not essential for diagnosis. In our experience, the malignant target sign is only rarely observed in the stomach.

##### Homogeneous Delayed Enhancement

**Figure 6:**
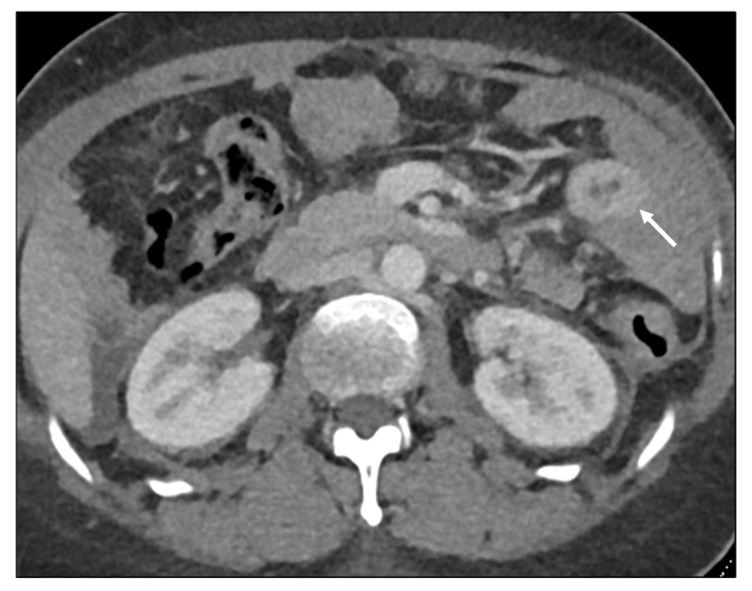
Homogeneous delayed enhancement. Axial contrast-enhanced CT. Metastasis to the descending colon (arrow) from undifferentiated gastric adenocarcinoma. Homogeneous enhancement at two minute-delayed phase. The layers are no longer discernible. This appearance correlates with the abundant desmoplastic reaction associated with linitis plastica.

A distinguishing imaging characteristic of LP-like metastases is their uniform enhancement during the 2-min delayed phase on contrast-enhanced cross-sectional imaging, at which point the layered structure is no longer visible (Figure 6). This finding reflects the pronounced desmoplastic reaction typical of LP, which is characterized by progressive enhancement [46].

##### Concentric Ring Pattern

**Figure 7:**
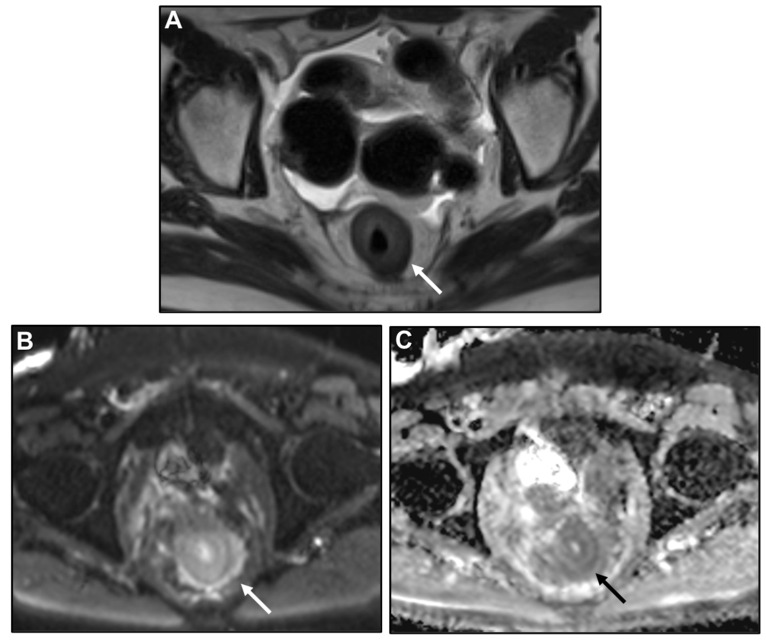
Concentric ring pattern on T2-weighted imaging (T2WI) and diffusion-weighted imaging (DWI). (**A**) MR image: Axial T2WI, (**B**) and (**C**) MR image: DWI and corresponding apparent diffusion coefficient (ADC). Shown is linitis plastica-like rectal metastasis from breast adenocarcinoma. Linitis plastica-like metastatic infiltration presents as mural thickening with preserved zonal anatomy. The stratified layers remain visible and their distinction appears accentuated. On both T2WI and DWI, the hallmark feature is a concentric ring pattern (arrow).

LP-like metastases to the GI tract typically present as a thickened segment with preserved zonal anatomy. Stratified layers will not only be preserved but, due to intervening tumoral tissue and desmoplastic reaction, the distinction between them will appear exaggerated. On magnetic resonance (MR), this may manifest as a characteristic concentric ring pattern on T2-weighted and diffusion-weighted images [47,48,49,50,51] (Figure 7).

##### Length

**Figure 8:**
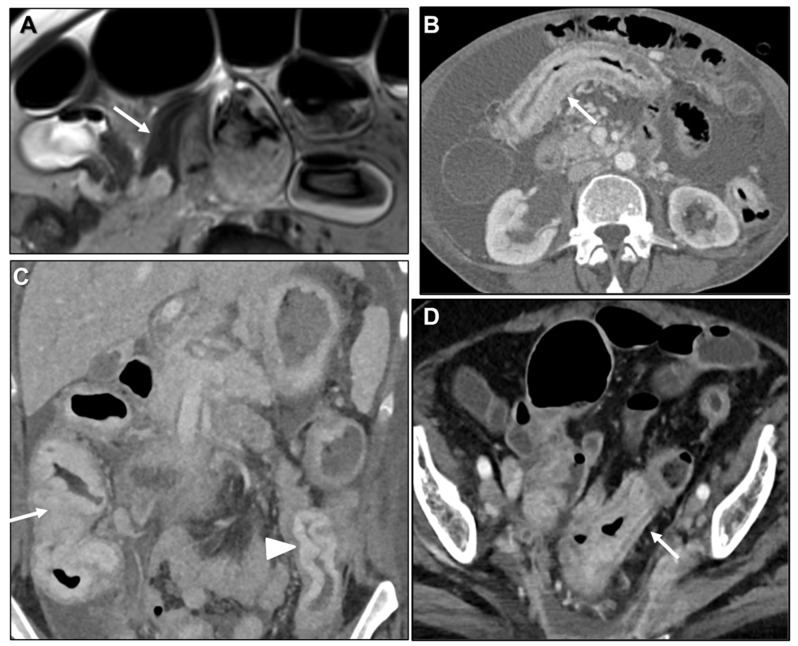
Different lengths of GI involvement. (**A**) MR image: Axial T2WI. Linitis plastica-like metastasis to small bowel (arrow) from invasive lobular carcinoma (ILC). (**B**) Axial contrast-enhanced CT (CE-CT). Linitis plastica-like metastasis to transverse colon (arrow) from ILC. (**C**) Coronal MPR CE-CT. Linitis plastica-like metastasis to ascending (arrow) and descending colon (arrowhead) from gastric adenocarcinoma. (**D**) Axial CE-CT. Linitis plastica-like metastasis to sigmoid colon (arrow) from ILC.

The extent of GI involvement can vary, ranging from long segments in the sigmoid, rectum, and anus to more localized, segmental disease in the small bowel and colon [46]. Similarly, gastric involvement may be focal but is more often diffuse (Figure 8).

**Figure 9:**
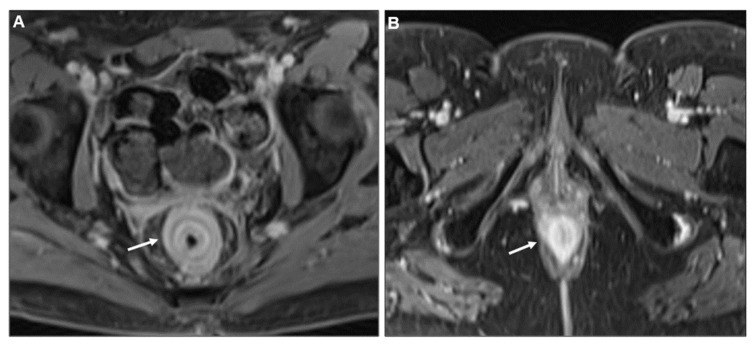
(**A**,**B**) MR images: Axial CE-GRE-T1WI. Linitis plastica-like metastasis to the rectum (arrow in (**A**)) from breast adenocarcinoma with extension to the anal verge (arrow in (**B**)), likely related to longitudinal intramural spread facilitated by peristaltic and antiperistaltic movements.

Kim et al. observed in their series of patients with primary colorectal carcinoma with LP appearance that when the rectum was involved, disease frequently reached the anal verge [24], probably secondary to the longitudinal intramural spread mentioned earlier. In our experience, this feature has also been observed relatively frequently in LP-like metastases involving the rectum (Figure 9).

#### 2.2.2. Associated Imaging Features

In addition to the key imaging features discussed above, other associated imaging features are commonly encountered when metastases to the gastrointestinal tract present with a LP pattern (Table 2).

##### Peritoneal Carcinomatosis

**Figure 10:**
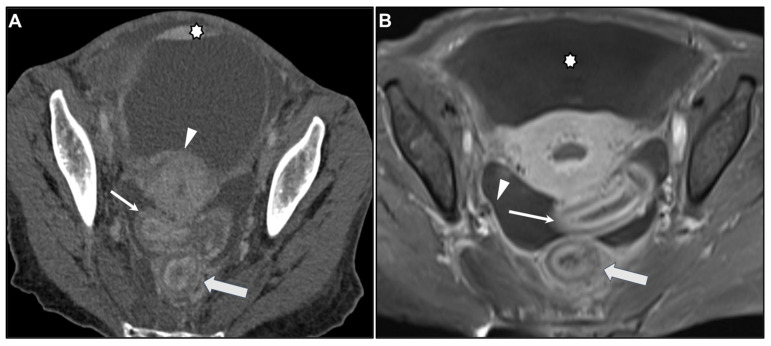
Concomitant peritoneal carcinomatosis (PC). (**A**) Axial contrast-enhanced CT. (**B**) MR image: Axial CE-GRE-T1WI. Linitis plastica-like metastases from invasive lobular carcinoma involving the rectum (thick arrows) and sigmoid colon (thin arrows). Concomitant PC is demonstrated by deposits over the bladder (* in (**A**)) and around the uterus (arrowhead in (**A**)), with ascites (* in (**B**)) and enhancing parietal peritoneum (arrowhead in (**B**)).

Concomitant peritoneal carcinomatosis (PC) is frequently observed, occurring in up to 50–80% of rectal LP-like metastases at diagnosis [44]. A characteristic presentation has been described in ILC with rectal LP-like metastases, featuring marked thickening of the bladder and vaginal walls and bilateral hydronephrosis, secondary to peritoneal involvement (Figure 10) [52].

##### Presacral Fat Tissue and Rectovaginal Septum Infiltration and T2-Weighted Imaging (T2WI) Hypointense Extramural Tumour Component

**Figure 11:**
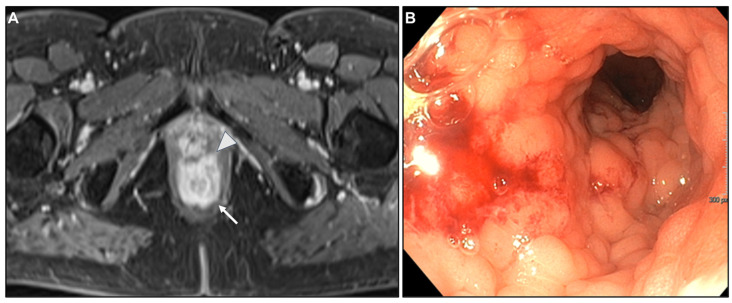
Infiltration of the rectovaginal septum. (**A**) MR image: Axial CE-GRE-T1WI. Linitis plastica-like rectal metastases (arrow) from breast adenocarcinoma with infiltration of the rectovaginal septum (arrowhead). (**B**) Corresponding colonoscopy reveals an extrinsic stenosis of the anus and rectum with rigid walls.

**Figure 12:**
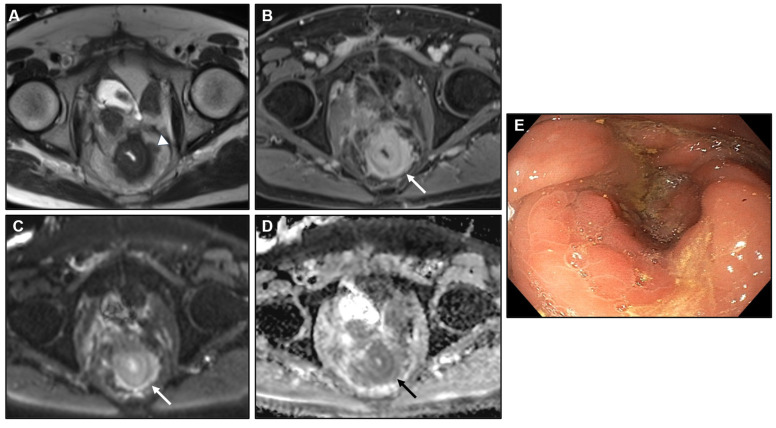
Extramural tumour component markedly hypointense on T2WI. (**A**) MR image: Axial T2WI. (**B**) MR image: Axial CE-GRE-T1WI. (**C**) MR image: DWI, (**D**) MR image: ADC. (**E**) Colonoscopy. Patient with bladder adenocarcinoma previously treated with surgery and chemotherapy. MRI showed an irregular contour with a strikingly hypointense extramural tumor component on T2WI (arrowhead in (**A**)), reflecting intense desmoplastic reaction. A concentric ring pattern of the rectum was evident, more pronounced on DWI (arrows in (**C**,**D**)), along with a malignant target sign (arrow in (**B**)). Colonoscopy revealed extrinsic rectal infiltration causing luminal occlusion, suspected to represent linitis plastica-like metastasis from bladder adenocarcinoma; however, biopsy results were negative. The patient subsequently underwent colostomy, during which peritoneal metastases were identified. The patient died shortly thereafter, and the diagnosis could not be definitively confirmed.

In anorectal LP-like metastases from ILC, two additional imaging features have been described: infiltration of the presacral fat and rectovaginal septum (Figure 11) [53], and the presence of an extramural tumor component that appears markedly hypointense on T2WI, likely reflecting intense desmoplastic reaction (Figure 12) [47]. However, these features are not exclusive to ILC, as we also observed a markedly hypointense extramural component on T2WI in rectal LP-like metastases from bladder carcinoma.

##### Intestinal Obstruction

**Figure 13:**
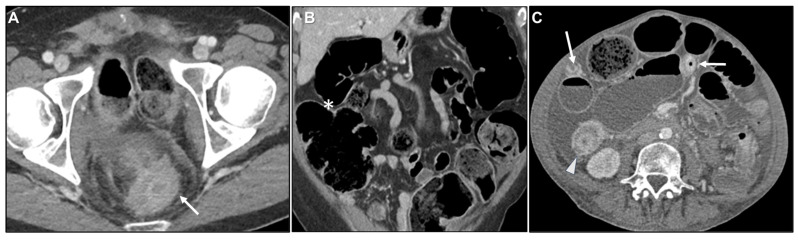
Bowel obstruction. (**A**) Axial contrast-enhanced CT (CE-CT). (**B**) MPR CE-CT. Patient with bladder adenocarcinoma with linitis plastica-like rectal metastasis (arrow in (**A**)) presenting as a colonic obstruction (* in (**B**)). (**C**) Axial CE-CT. Patient with linitis plastica-like metastases from invasive lobular carcinoma to small bowel (arrows) and ascending colon (arrowhead) resulting in obstruction at multiple levels.

**Figure 14:**
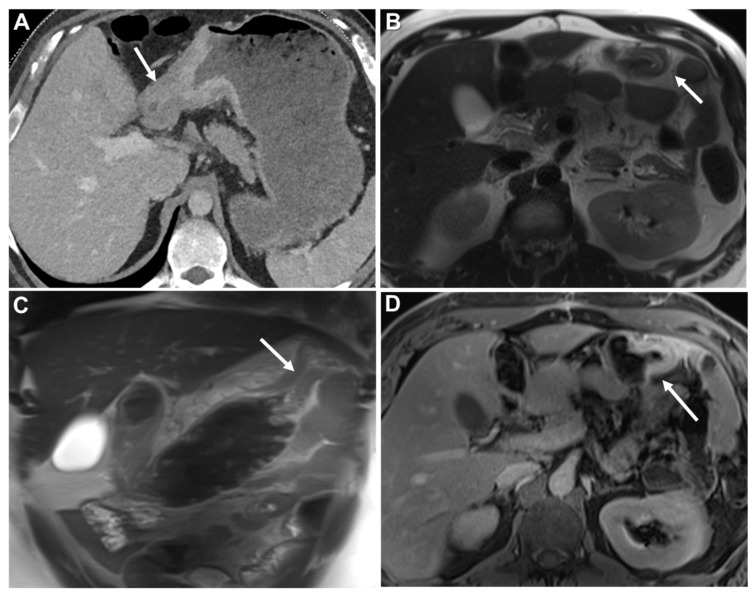
Bowel obstruction. (**A**) Axial contrast-enhanced CT. (**B**) MR image: Axial T2WI. (**C**) MR image: Coronal T2WI. (**D**) MR image: Axial CE-GRE-T1WI. Patient with poorly differentiated gastric adenocarcinoma presenting with a linitis plastica (LP) appearance (focal form) (arrow in (**A**)). The patient achieved complete remission but, two years after diagnosis, developed a colonic obstruction showing a LP-like metastasis at the transition point in the distal transverse colon (arrows in (**B**–**D**). The LP-like metastasis, most evident on axial images, is characterized by concentric mural thickening and a malignant target sign following contrast administration (arrow in (**D**)). Surgery revealed additional peritoneal deposits, which were not detectable on imaging. Immunohistochemistry of the biopsy confirmed metastasis to the transverse colon from the gastric adenocarcinoma.

ILC is the most common cause of bowel obstruction resulting from metastatic involvement of the intestinal wall [54]. Moreover, any intestinal LP-like metastasis has the potential to cause obstruction (Figure 13). Colorectal obstruction caused by LP-like metastases has, in some cases, been reported as the initial manifestation of gastric adenocarcinoma [55,56] (Figure 14).

### 2.3. Diagnostic Challenges

LP-like metastases to the GI tract present a diagnostic challenge. Reaching an accurate diagnosis requires integrating patient information, including prior clinical history, with both imaging and pathological findings.

#### 2.3.1. Anamnesis

Several key points should be considered to avoid misinterpretation when assessing GI abnormalities suspicious for malignancy on cross-sectional imaging.

Considering that the interval between the diagnosis of the primary tumor and the development of GI metastases can be prolonged (up to 30 years in breast carcinoma) [57], this crucial aspect of the patient’s oncologic history may at times be overlooked or lost. Therefore, obtaining a thorough medical history is essential for an accurate diagnosis. Furthermore, the rarity of GI metastases, combined with the possibility that they may represent the sole site of disease recurrence without evidence of active disease elsewhere, can contribute to a low index of suspicion [58].

**Figure 15:**
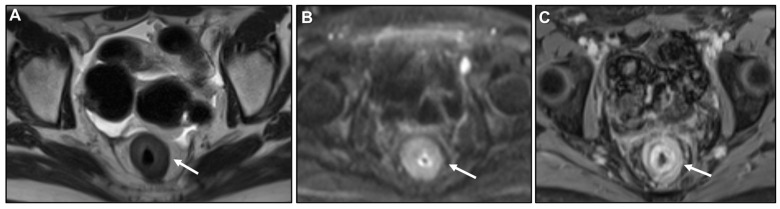
(**A**) MR image: Axial T2WI. (**B**) MR image: DWI. (**C**) MR image: Axial CE-GRE-T1WI. Female patient with bladder adenocarcinoma, previously treated with surgery and chemotherapy. Follow-up MRI revealed rectal wall thickening, showing a concentric ring pattern on T2-weighted imaging (arrow in (**A**)) and a malignant ring sign after intravenous contrast administration (arrow in (**C**)). The concentric ring pattern is not visible on diffusion (arrow in (**B**)). Initially suspected to be linitis plastica-like rectal metastasis from the known bladder primary, immunohistochemistry ultimately identified the origin as an occult breast adenocarcinoma.

In addition to LP-like GI metastases from a known primary tumor, a small proportion of cases (3–5%) arise from an unknown primary malignancy [59]. In such instances, immunohistochemistry (IHC) is essential for identifying the primary site; however, recognizing an LP pattern on cross-sectional imaging can also be highly valuable in narrowing the differential diagnosis and guiding the search for the primary tumor (Figure 15).

#### 2.3.2. Clinical Presentation

LP-like metastases to the GI tract may initially be clinically silent. While they can cause intestinal obstruction, as previously described, they are more often associated with nonspecific GI symptoms that may easily be attributed either to ongoing cancer therapy or to other benign gastrointestinal conditions [60,61].

#### 2.3.3. Imaging Findings

LP-like GI metastases can be difficult to detect because of their infiltrative growth pattern. On CT, they often present as smooth bowel wall thickening, which may be misinterpreted as normal peristaltic activity [37].

The frequent coexistence of PC adds another layer of diagnostic difficulty. While metastases affecting retroperitoneal bowel segments are relatively straightforward to identify, distinguishing intraperitoneal involvement from peritoneal deposits can be challenging. The absence of peritoneal disease and ascites may support a diagnosis unrelated to peritoneal dissemination; however, confidently excluding peritoneal deposits on cross-sectional imaging remains extremely difficult. The differentiation becomes more conspicuous thanks to the key imaging features previously described. In cases of PC with GI tract infiltration secondary to serosal deposits, tumor infiltration progresses centripetally, with the tumor burden predominantly affecting the superficial layers and the zonal anatomy is not preserved; thus, the malignant target sign and concentric ring pattern are absent.

#### 2.3.4. Pathology

Histological features alone do not always allow differentiation between a LP-like GI metastasis and a peritoneal deposit invading the GI wall. In such cases, imaging plays a crucial role in distinguishing serosal deposits (peritoneal carcinomatosis) from subserosal metastases. In clinical practice, GI metastases often remain pathologically unconfirmed (Figure 12). They typically appear at an advanced stage of disease [62], when patients are in poor clinical condition and less likely to undergo endoscopic evaluation. Even when performed, endoscopy may yield false-negative results, as LP-like GI metastases are predominantly submucosal [63] and frequently spare the mucosa. Consequently, superficial biopsies may be negative, making deep and extensive sampling necessary when LP-like GI metastases are suspected [64]. Obtaining a complete pathological specimen is also challenging: many exploratory laparoscopies are terminated immediately due to widespread disease. Moreover, even when resection is technically feasible, metastasectomy does not improve prognosis, and surgery is generally reserved for palliation in cases of obstruction or perforation.

### 2.4. Differential Diagnosis

The differential diagnosis for conditions appearing as LP can be separated into two main categories: malignant and benign causes.

#### 2.4.1. Malignant Causes

**Figure 16:**
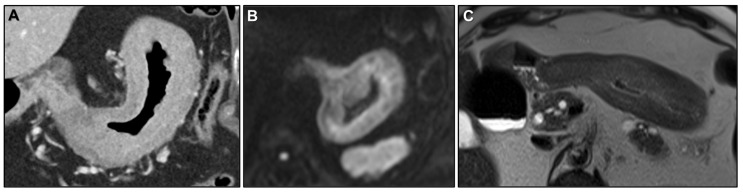
(**A**) Coronal MPR contrast-enhanced CT. (**B**) MR image: DWI. (**C**) MR image: Axial T2WI. Patient with invasive lobular carcinoma (ILC) on chemotherapy presenting with malaise. Imaging shows a diffusely thickened, contracted stomach with a linitis plastica (LP) pattern and loss of gastric folds. The differential diagnosis included primary gastric adenocarcinoma versus LP-like ILC metastasis. Immunohistochemistry after deep biopsy confirmed gastric metastasis from ILC.

The main differential diagnosis is primary GI adenocarcinoma with an LP appearance, most often undifferentiated or poorly differentiated. Immunohistochemistry (IHC) is decisive in this setting, as conventional histology alone may be insufficient. For example, invasive lobular carcinoma (ILC) may contain numerous signet ring cells, and when combined with its typical mucosa-sparing pattern of spread, it can mimic either metastatic disease to the stomach or a primary gastric LP tumor (Figure 16).

**Figure 17:**
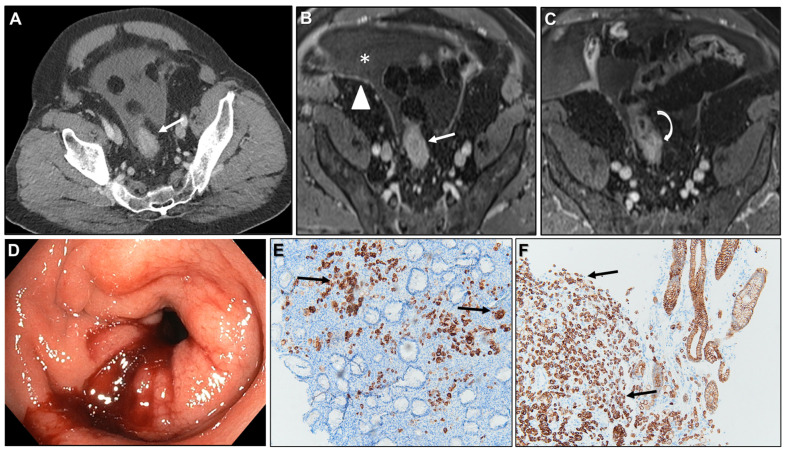
(**A**) Axial contrast-enhanced CT. (**B**,**C**) MR images: Axial CE-GRE-T1WI. (**D**) Colonoscopy. (**E**) 40X Cytokeratin 7 (CK7) immunohistochemistry (IHC) staining tumour cells within the sigmoid lesion. (**F**) 40X CK7 IHC staining tumour cells within the gastric primary tumour. Patient with signet ring cell gastric adenocarcinoma treated surgically, later developing peritoneal carcinomatosis (PC). On follow-up CT, a linitis plastica-like sigmoid lesion was identified (arrow in (**A**)), demonstrating a malignant target sign (arrow in (**B**)) and abrupt mural thickening at the transition point (curved arrow in (**C**)). Ascites (* in (**B**)) and parietal peritoneal deposits (arrowhead in (**B**)) were also present. Colonoscopy (**D**) showed edematous, erythematous mucosa with a stenosis 20 cm from the anal verge, but no visible mass. Deep biopsies were performed, and IHC confirmed metastasis from gastric adenocarcinoma. Atypical cells infiltrating the rectal lamina propria (with mucosal involvement in this case) stained positive for CK7 in both the sigmoid lesion (arrows in (**E**)) and the gastric primary tumor (arrows in (**F**)). CK7, a membranous/cytoplasmic keratin marker expressed in many epithelia and epithelial neoplasms, is typically positive in upper gastrointestinal tumors but negative in colorectal carcinomas.

**Figure 18:**
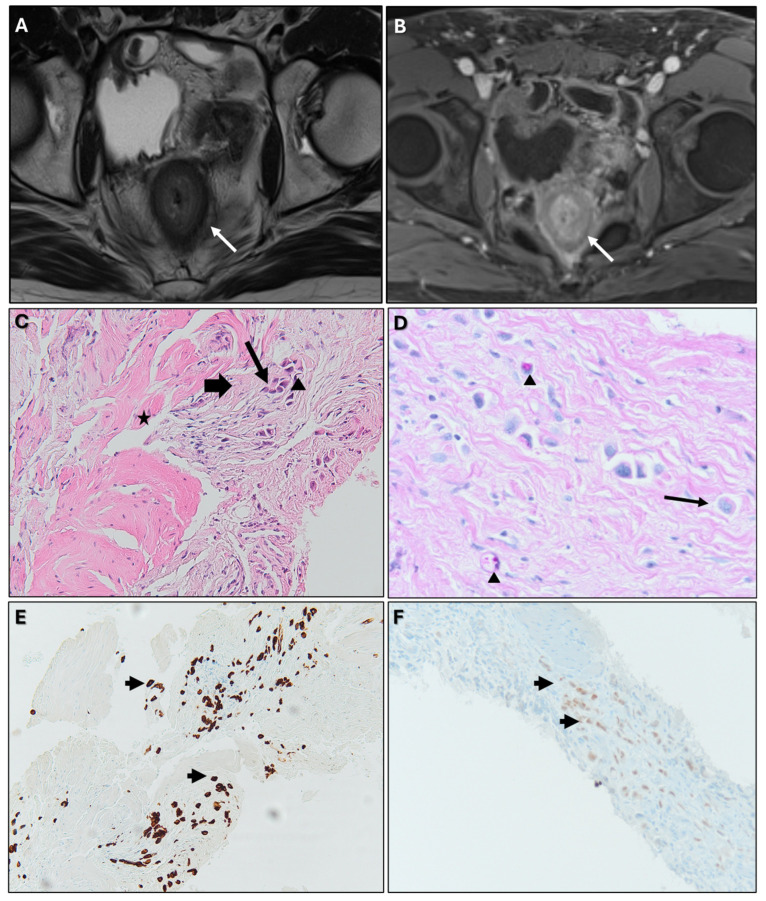
Patient with bladder adenocarcinoma (plasmacytoid variant with independent cells), treated with surgery and chemotherapy, presenting with suspected linitis plastica (LP)-like rectal metastasis on follow-up MRI. (**A**) MR image: Axial T2WI: concentric ring pattern (arrow). (**B**) MR image: Axial CE-GRE-T1WI: malignant target sign (arrow). (**C**) 100X Hematoxylin and eosin (H&E: tumour infiltration of the rectal muscularis propria. Normal muscle cells (*) are surrounded by desmoplastic tissue (thick arrow) containing atypical cells (thin arrow) and occasional signet ring cells (arrowhead). (**D**) 100X PAS special staining: positive for mucin, highlighting signet ring cells (arrowheads) and the plasmacytoid morphology of tumour cells (thin arrow). Plasmacytoid urothelial carcinoma is a variant of infiltrating urothelial carcinoma, showing morphologic and immunohistochemical resemblance to plasma cells. (**E**) 40X CK7 IHC: tumour cells (arrows) show membranous/cytoplasmic positivity. CK7 is typically expressed in urothelial carcinoma and negative in colorectal carcinoma. (**F**) 40X Gata3 IHC: tumour cell nuclei (arrows) are positive. GATA3 is expressed in most urothelial carcinomas (primary and metastatic) but not in rectal carcinoma.

Therefore, IHC plays a pivotal role in diagnosis: metastatic ILC typically shows positivity for CK7, ER, PR, and GCDFP-15, and negativity for vimentin, whereas CK20 and CEA are almost invariably expressed in primary gastrointestinal tumors but absent in breast carcinomas [65,66]. Nonetheless, the contribution of CT and MRI in supporting the diagnosis of secondary LP should not be underestimated. While the possibility of a second primary tumor exists, the detection of an LP pattern on imaging should first raise suspicion for metastasis (Figure 17 and Figure 18).

Other primary tumors that may present with an LP appearance include both Hodgkin and non-Hodgkin gastric lymphomas [67,68]. Distinguishing imaging features of gastric lymphoma are the relatively mild contrast enhancement and partial preservation of gastric distension, owing to the absence of a desmoplastic response [69]. Kaposi’s sarcoma has also been reported, albeit rarely, with an LP-like presentation [70,71].

Together, the histological and IHC profile confirmed that the rectal lesion represented metastasis from the primary bladder tumor.

#### 2.4.2. Benign Causes

Several benign conditions can mimic gastric LP-like metastases, including infections such as CMV [72], syphilis [73], and tuberculosis [74], as well as inflammatory processes like sarcoidosis, radiation-induced changes, and acute Crohn’s disease [74,75] (Table 3).

These conditions also demonstrate a preserved layered wall pattern [76,77]. The presence of the benign target sign, characterized by a hypoenhancing edematous submucosa, can facilitate diagnosis. Additional supportive imaging features include inflammatory changes in the surrounding fat and the involvement of a long, solitary segment, both of which are more typical of benign processes [20]. In contrast, short and multifocal segments are more suggestive of GI metastases. However, the length criterion should be applied with caution, as both short and long segments may be affected [78]. Chronic inflammatory bowel disease with fibrotic changes and homogeneous wall enhancement on delayed-phase imaging may be difficult to distinguish, particularly since malignant transformation can also occur in this setting [79].

Despite the challenge, establishing the correct diagnosis is essential, as management and prognosis differ significantly. Surgical resection is the standard approach for non-metastatic primary gastric, small bowel, and colorectal carcinomas, but it may also be considered in selected cases of solitary, resectable GI metastases, particularly in the stomach [80] or rectum [42]. Such cases warrant thorough assessment by a multidisciplinary tumor board, as the decision depends on both the patient’s clinical condition and the characteristics of the primary tumor [42]. In the palliative setting, international guidelines recommend endocrine systemic therapy for breast cancer metastases [81,82], while radiotherapy can be considered in selected cases of rectal involvement. 

## 3. Conclusions

Although LP-like GI metastases are rare, their recognition is clinically significant, and awareness of their characteristic features on cross-sectional imaging is essential for accurate diagnosis.

While immunohistochemistry (IHC) remains the definitive diagnostic tool, the supportive role of CT and MR in identifying LP-like metastases should not be underestimated. Radiologists play a key role in raising suspicion, recommending deep and repeated biopsies given the frequent sparing of the mucosa.

Furthermore, in cases of an unknown primary tumor, recognition of the LP pattern can provide valuable clues to the potential site of origin.

## Figures and Tables

**Table 1:** Key imaging features of metastases to the gastrointestinal tract with linitis plastica appearance.

Key Imaging Features
Malignant target sign
Delayed homogeneous enhancement
Concentric ring pattern

**Table 2:** Associated imaging features of metastases to the gastrointestinal tract with linitis plastica appearance.

Associated Imaging Features
Peritoneal carcinomatosis
Presacral fat tissue and rectovaginal septum infiltration
Extramural tumour component markedly hypointense on T2WI
Intestinal obstruction

**Table 3:** Differential diagnosis for linitis plastica appearance: malignant and benign conditions.

Malignant	Benign
Primary adenocarcinoma	Infectious (CMV, syphilis, tuberculosis)
Hodgkin and non-Hodgkin gastric lymphoma	Inflammatory diseases (sarcoidosis, acute Crohn’s disease, radiation induced changes)
Kaposi’s sarcoma	Chronic inflammatory bowel disease

## Data Availability

No new data were created or analyzed in this study. Data sharing is not applicable to this article. All images are original from our own clinical cases.

## Abbreviations

The following abbreviations are used in this manuscript:

ADC	apparent diffusion coefficient
CE	contrast enhanced
CT	computer tomography
CK7	cytokeratin 7
DWI	Diffusion-weighted imaging
GI	gastrointestinal
GRE-T1WI	gradient recalled echo T1-weighted images
H&E	hematoxylin and eosin
IHC	immunohistochemistry
ILC	invasive lobular carcinoma
LP	linitis plastica
MP	muscularis propria
MPR	multiplanar reconstruction
MR	magnetic resonance
SB	small bowel
PC	peritoneal carcinomatosis
T2WI	T2-weighted imaging
